# DarwinGSE: Towards better image retrieval systems for intellectual property datasets

**DOI:** 10.1371/journal.pone.0304915

**Published:** 2024-07-01

**Authors:** João António, Jorge Valente, Carlos Mora, Artur Almeida, Sandra Jardim

**Affiliations:** 1 Techframe-Information Systems, SA, São Domingos de Rana, Portugal; 2 Smart Cities Research Center, Polytechnic Institute of Tomar, Tomar, Portugal; Mirpur University of Science and Technology, PAKISTAN

## Abstract

A trademark’s image is usually the first type of indirect contact between a consumer and a product or a service. Companies rely on graphical trademarks as a symbol of quality and instant recognition, seeking to protect them from copyright infringements. A popular defense mechanism is graphical searching, where an image is compared to a large database to find potential conflicts with similar trademarks. Despite not being a new subject, image retrieval state-of-the-art lacks reliable solutions in the Industrial Property (IP) sector, where datasets are practically unrestricted in content, with abstract images for which modeling human perception is a challenging task. Existing Content-based Image Retrieval (CBIR) systems still present several problems, particularly in terms of efficiency and reliability. In this paper, we propose a new CBIR system that overcomes these major limitations. It follows a modular methodology, composed of a set of individual components tasked with the retrieval, maintenance and gradual optimization of trademark image searching, working on large-scale, unlabeled datasets. Its generalization capacity is achieved using multiple feature descriptions, weighted separately, and combined to represent a single similarity score. Images are evaluated for general features, edge maps, and regions of interest, using a method based on Watershedding K-Means segments. We propose an image recovery process that relies on a new similarity measure between all feature descriptions. New trademark images are added every day to ensure up-to-date results. The proposed system showcases a timely retrieval speed, with 95% of searches having a 10 second presentation speed and a mean average precision of 93.7%, supporting its applicability to real-word IP protection scenarios.

## 1 Introduction

Over time, information has become a fundamental basis for the evolution of society, with a growing awareness that its storage and distribution are of paramount importance for its sustained growth. Social, intellectual and technological evolution has led to the methods of storing and transmitting information to progress steadily, becoming increasingly efficient. Currently, and almost globally, access to information is almost instantaneous, boosting the creation of more and more knowledge among the world’s population. This trajectory led to an exponential growth in the volume of data and information created and stored every day, making the search for information an arduous task, if not impossible, when not supported technologically. In this context, Information Retrieval Systems (IRS) are extremely important, as they help users to find relevant information from the large amount of available data.

Information Retrieval (IR) finds applications in several areas, among which are Digital Libraries, Search Engines, Media Search—that can involve text, image, music, speech and video retrieval processes—and Information filtering, such as recommendation systems. As a specific sub-area of Information Retrieval [1], image retrieval has been of great interest to the scientific community since the 1970s. Image retrieval is the process of searching a large database of digital images for those that are most similar to a given query image [2–4].

Image retrieval systems can follow two approaches: query-by-text, where queries are texts and targets are images, and query-by-example, where both queries and targets are images [5]. In a query-by-text image retrieval system, known as annotation-based image retrieval (ABIR), images are usually annotated by words. Sometimes, as a result of compression processes or the occurrence of human error, these annotations can be lost. Another difficulty stems from the wide variety of image annotation standards, making it difficult to define a common ontology. In a query-by-example approach, although annotations can be used but are not required, which allows to overcome the difficulties of text-based image retrieval [6]. In this case, the search is performed based on the content of the image, making these systems known as Content-Based Image Retrieval (CBIR) systems [7], where images are compared and classified by similarity. In CBIR systems, images are represented by their visual features that can be global, such as shape, texture, and color, or local, such as edges, points, and image patches. These visual contents are extracted and described by vectors of multidimensional features and the image retrieval is done by calculating the similarities/distances between the feature vectors of the query image and the database images [8–10]. Some CBIR systems contain a user-relevance feedback mechanism to refine the query results, generating perceptually and semantically more meaningful retrieval results [11–13].

With the increase in computational processing capacity, accompanied by the decrease in memory prices, processing large amounts of data has become a reality, for what the development of systems capable of processing it efficiently is a necessity. Regarding image retrieval, there are several areas of application, where the volume of images currently available is enormous, allowing the developed systems to undergo rigorous validation and testing processes. Among the different areas of application of CBIR systems are medical imaging [14–22], criminal investigation [6, 23–25], biology [26–29], digital libraries [30–32] and intellectual property [6, 33–37].

**Fig 1:**
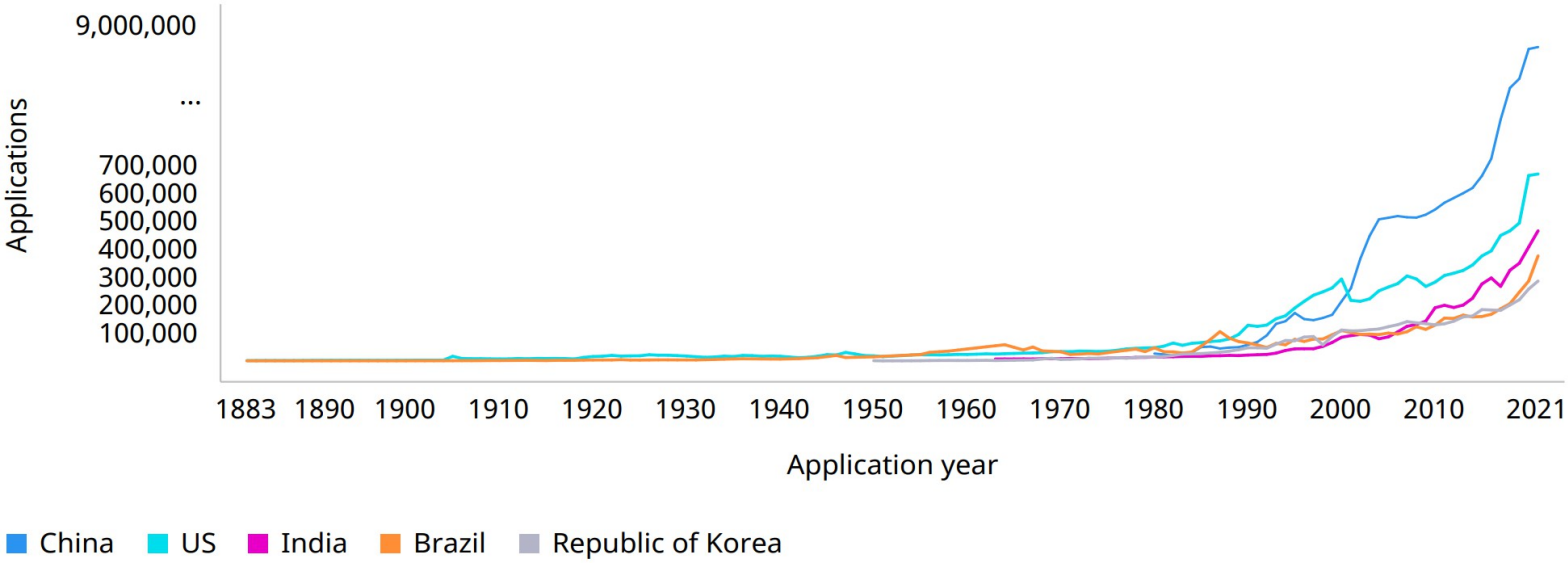
Trend in trademark applications for the top five offices, from 1883 to 2021 [40].

Intellectual property is an integral part of intellectual capital [38, 39]. A trademark is a legal right of intellectual property, which may assume several forms, such as a symbol, an image, a logo, or a word. In general, images or logos are used, which can be combined with words or slogans, so that the products or services offered are more easily identified by customers and associated with companies. The registration of a trademark addresses as one of its objectives the protection of its value and of the products and services made available by the company. Additionally, the creation of a trademark aims to boost its growth, disseminating it in a simple way through different channels and promoting customer loyalty. For a trademark to be legally protected, it must be registered within an international, regional or national intellectual property organization, such as the World Intellectual Property Office (WIPO). Depending on the type of products or services associated with a trademark, its registration may be related to different classes. Generally, the Nice Classification is used, an international standard, where each trademark is classified in one or more of the 45 defined Nice classes depending on the type of products or services that will be marketed under it [40]. Fig 1 illustrates the evolution of trademark applications in the five offices with the highest volume of applications worldwide, between 1983 and 2021.

According to the latest WIPO report, the number of trademark registrations worldwide has seen a double-digit increase in 2021, reaching 73.7 million, which corresponds to an increase of 14.3% compared to the number of trademarks registered in 2020. This reality forces intellectual property and law officers to analyze large amounts of data in search of registered trademarks that may be similar in text, phonetic, or image components, to any new trademark whose registration is requested, to guarantee that there are no duplicate or similar trademarks that may induce the consumer into confusion. This search is done manually, following what is specified in the Vienna Classification System, a commonly used method of identifying the graphic components in a trademark, which, in addition to being very time-consuming, translates into a process with inconsistent results, given the subjectivity inherent of the human factor in classifying the image components [41, 42]. This increase in the number of trademarks to be searched also promotes the occurrence of errors that may lead to the violation of the legal rights of registered trademarks.

**Fig 2:**
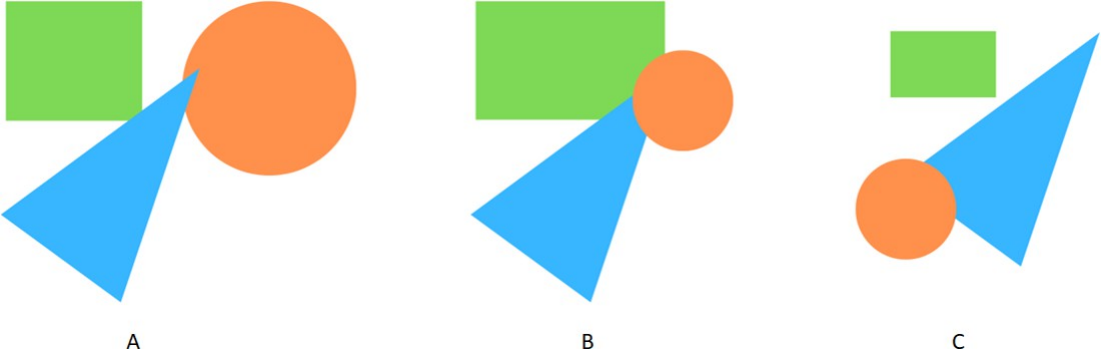
Human perception of similarity. A: Image composed of three objects. B: Same objects, different layering and scale but similar position. C: Same objects, different scaling and position.

Faced with this reality, the expectation that the number of trademark applications will continue to increase over the years, and the awareness of the importance of trademark protection, both for companies and for society in general, some efforts have been made to develop trademark image retrieval systems. Despite the existence of some proposals for trademark image recovery systems, to the best of our knowledge, there is still none whose performance allows its use in a real context. Indeed, the application of CBIR systems to the area of Intellectual Property is particularly challenging, since it is intended to model the human perception of abstract images, where shape is crucial [43], and to identify and identify those that may be similar from the point of view of human perception. To better understand the complexity of creating an automatic system capable of identifying similar logos, in the same way that a human being would, let us consider the hypothetical logos illustrated in Fig 2. According to the human perception system, the images of Fig 2A and 2B are considered more similar, even though the image of Fig 2C is composed exactly by the same objects as the image of Fig 2A, being only in different locations [41].

In the context of Intellectual Property, an automatic image retrieval system must, in addition to evaluating the global shape of an image, consider the variations that may occur in the objects that compose it, such as rotations, changes of dimensions, inversions, noise and overlaps.

Although there have been considerable advances, mostly concentrated on the ability to process large volumes of images and on the use of deep learning models, CBIR systems still have several problems, namely concerning their effectiveness and reliability [44–50], which highlights a gap between the results achieved by existing solutions and those expected from a system to be used by the market, particularly in the area of Intellectual Property [41], where lack of precision can lead to lawsuits.

An image retrieval system with application in the area of Intellectual Property must a) consider all possible human interpretations of a trademark image; b) have a processing time allowing search in large image repositories in a short time; c) have zero tolerance, not omitting results that may be considered similar by humans [41]. These three characteristics, indispensable in a trademark image retrieval system for real use, are the main limitations that we found in the systems proposed so far.

Regarding human perception modeling, and given the complexity of interpreting abstract images, except for the Trademark Archival and Registration system (STAR) [51], the proposed solutions do not consider the different interpretations that a trademark image can have. In fact, most of these systems are based on low-level features [6, 35, 52], where the interpretation of the image content can result from its global analysis to its analysis by segments/regions or contours.

Regarding processing time, we found that the approaches that have been proposed are based on quantities of images in the thousands, with no particular importance being given to the efficiency of the search process. Resorting to image analysis based on low-level features require high processing times, which limits the use of these approaches in a real context. In the field of Intellectual Property, where the volume of images grows daily, the design of a CBIR must consider the efficiency of the system as a requirement, enabling its adoption by the market [46, 53]. One of the ways to increase the efficiency of a CBIR system when searching a large database is to divide it into different groups and perform sub-searches. In the case of brand images, this division can be carried out using the Nice Classification, where the registration of a brand image implies its classification in one of the 45 possible Nice classes, which refer to the general market or type of services provided by the underlying business.

Ensuring the reliability of a CBIR system with application in the area of Intellectual Property requires some degree of user verification to be possible at any given time, as well as zero-tolerance for false negatives, since the breach of these rules may lead to serious legal actions against the offending trademark.

Addressing the challenges mentioned above, this paper details a new graphical search engine designed for use with Intellectual Property data, and outlines the additional modules that grant scalability and gradual optimization of search parameters. The proposed system, code-named Darwin Graphical Search Engine, is capable of providing accurate results regardless of the complexity and size of the dataset and submitted query. To achieve a high degree of retrieval performance, we propose several design choices and additional components that are aimed at fine-tuning CBIR systems for IP problems. As such, the methodological contributions of our paper are:

- A real-time image processing mechanism to extract several layers of meaningful features from any image input. We propose a combination of CNN-Based Feature Descriptions and Sentence Embedding Model Descriptors to achieve a high degree of sensitivity to the image contents.
- A background clustering mechanism that automatically sorts visually compatible images into several predetermined groups. These groups were manually analyzed for visual similarities, and the most important characterizing features of each cluster were recorded, enabling faster retrieval by eliminating the need to exhaust the entire dataset when confronting images.
- The introduction of a relevance feedback module, allowing users to approve/disapprove individual images in the result set. These changes are privately recorded and modelled with a novel approach to determine whether the corresponding search fit the user’s expectations, prompting changes to the hyper parametrization functions if not.
- A parallel searching mechanism, utilizing Facebook AI Similarity Search (FAISS) indexes to quickly analyze every image pair in regard to their sentence embeddings. Traditional, cluster-based searches are then expanded with high-confidence hits achieved through this method, improving the zero-tolerance capabilities of the system against false negatives.

### Document organization

This document is divided into sections, subsections, and details. **Section 2—Related work** describes related work and different state-of-the-art approaches used for content-based image retrieval systems. In **Section 3—Methodology**, a simplified presentation of the system is provided. In **Section 4—Image comparison module**, the method by which results are qualified and presented is explained. In **Section 5—Reinforcement Learning Module**, the reinforcement learning implementation used for gradual result improvement is outlined in detail. In **Section 6—Growth module**, a cluster of algorithms of recurring processing used in the context of keeping up-to-date information is described. In **Section 7—Results**, a series of five distinct query results are presented and interpreted regarding the individual impact of the proposed components. In **Section 8—Scientific and technological relevance**, the collection of the aforementioned systems is analyzed and interpreted from a success metrics point of view, alongside any relevant contributions to the relative areas of expertise. Finally, in **Section 9—Conclusion**, a more personal takeaway on the project’s impact is provided, as well as some general directions for future expansion of the system.

## 2 Related work

In the past half-century, science has seen the birth and growth of the Information Retrieval area of research, but only more recently have significant leaps in the depth, complexity and performance of these systems been observed, credit to, in part, the rise of Big Data as a concept that redefined the expectations of modern technological systems.

### Conventional feature extraction approaches

One of the fundamental steps of a CBIR system is the extraction of relevant information from the visual content of images. This information, designated as image features, is stored as feature vectors, also known as image signatures, with which is calculated the similarity between the query image and the images stored in the repository. Image features can be global, describing its entire visual content, such as color, spatial information, shape and texture, or local, describing patterns visually identifiable in image regions, such as edges and points.

Prior to ML-driven approaches, extracting relevant features from image data was often achieved with smart overlaying of several image processing techniques intended to target specific, handcrafted features from graphical information, to be extracted and evaluated to a degree of similarity.

Many of the initially proposed approaches focused on the extraction of global features [54–56], where color was one of the most explored features [57–61], given its importance in the human ability to distinguish objects. Even though several works have been presented over the years that use only one global characteristic to retrieve images [62–66], it is consensual that the results achieved fall short of what is desirable, since, in general, the images have several pictorial characteristics [67]. To achieve more accurate results, approaches emerge where more than one resource is combined, such as color and texture [68–70], color and spatial information [71], or color, texture and shape [72–74]. Despite this process, called resource merging, obtaining better performances, the accuracy of the recovery is still not satisfactory. This is mainly due to the fact that these characteristics change under scaling and rotation. For this reason, local features have gained popularity since they are invariant to geometric transformations, being more reliable in various conditions.

Among the most common local descriptors used in CBIR systems are Scale-invariant Feature Transform (SIFT) [75], Sped-up Robust Features (SURF) [76], Local Binary Pattern (LBP) [77], Histogram of Oriented Gradient (HOG) [78], Co-occurrence Histogram of Oriented Gradient (CoHOG) [79], Binary Robust Invariant Scalable Keypoints (BRISK) [80], and Features from Accelerated Segment Test (FAST) [81].

It is widely accepted that the Scale-Invariant Feature Transform (SIFT) algorithm, was one of the main driving forces behind conventional local feature extraction efforts. Despite being a robust technique against scale changes and rotations, its effectiveness decreases for images with noise or complex backgrounds [82], presenting a low performance in high dimensions [83]. BRISK comes up with the objective of overcoming the limitation of SIFT in the extraction of features in images with low illumination and poor location of key points. However, it has not been tested for large-scale unlabeled datasets, therefore its performance in these situations is not known. Also inspired by SIFT, SURF was created, where the limitation of operating in high-dimensional contexts of the first one is overcome, also being robust to lighting variations [84, 85]. However, this advantage entails a disadvantage compared to SIFT, since its performance in resource calculation is lower. Furthermore, unlike SIFT, SURF is not rotation invariant. Some approaches have been proposed that combine SIFT and SURF [86, 87], with which it is possible to increase the accuracy and efficiency of the image retrieval process.

By segmenting the images and calculating the distribution of local intensity gradients, the local HOG descriptor extracts the shape of objects present in the images. Being a robust technique in relation to brightness variations and the presence of shadows, it has been explored by some researchers, alone or combined with other descriptors, [88–90], demonstrating good results, particularly in object recognition. However, the use of HOG prevents multispectral images from being directly used.

Despite the significant advances in the field of Machine Learning, particularly in Deep Learning and its application to image processing [91], including image retrieval, several approaches based on more conventional techniques continue to be proposed. Some of these approaches use the feature fusion process to increase the accuracy of the system, such as the one proposed by Alsmadi. This approach creates a feature repository, containing color signatures and shape and texture features. Color signatures are extracted in the YCbCr color space through the Discrete Wavelet Transform and the Canny edge histogram, and shape and texture features are extracted in the RGB color space using neutrosophic clustering and Canny edge algorithms, and gray-level co-occurrence matrix, respectively [56]. Although having greater precision compared to previous proposals, the method proposed by Alsmani presents a critical cooling process, as well as a longer processing time. Another example of this technique is found in the work proposed by Ashraf *et al*. that fuse the color and texture features, extracted through color moments, and Gabor Wavelet filters and the Discrete Wavelet Transform, respectively [92]. Also in this case, the increase in precision comes with a high computational cost, given the large size of the feature vector.

Recently, Bu *et al*. presented a content-based image retrieval method that combines color, texture and shape features [93]. Authors use color autocorrelogram for color extraction while texture features are extracted using uniform magnitude local binary pattern and Gabor local correlation. For the extraction of shape features, the Machine Learning algorithm Support Vector Machine (SVM) is used. Although SVM presents good results for complex data, its performance is low for large-scale databases. Given this limitation, the authors tested the proposed method in repositories with a maximum of 1000 images.

Another recent work is the one proposed by Fadaei, who describes a Dominant Color Descriptor method [94], where the Canny algorithm is used to extract edges from images. After the dilation of the edges by a disk-shaped structuring element, the pixels belonging to them are differentiated from those that do not belong through the attribution of weights. With this procedure, the dominant color descriptor is created, resulting from the higher weighting of the most informative pixels, to the detriment of those belonging to regions with few color variations. In addition to presenting a greater image recovery time than several methods with similar precision values, such the proposed by Alsmadi [56], the approach also has the limitation of the need to calculate the size of the structuring element separately for each dataset.

In the context of Industrial Property, one of the first approaches considers trademark images as a whole. Following this approach, the system developed by Kato [7] is a pioneer, having appeared several variants where the increase in results accuracy is not considerable.

Another frequent approach, instead of processing the image as a whole, considers it composed of a discrete set of components/objects, which are compared individually, calculating the similarity of images using the partial similarities of the components. Following this methodology, in 1996, Wu created the STAR [51] system, which comprises two phases: a manual indexing phase and an automatic indexing phase. The complementing of the two processing phases allows for a very comprehensive characterization of the images, producing highly accurate results. The major limitation of this system lies in the need for the intervention of a human operator, which leads to a slow process, as well as the susceptibility to errors. To reduce, or even eliminate, the need for human intervention, several variants of this methodology have been proposed, such as the ARTISAN system, created by Eakins *et al*. in 1998 [95], and its improved version, developed in 2001 [96].

In the last decade, some image retrieval systems were proposed with applications in the field of Intellectual Property. Chen *et al*. propose a conventional method in which several image processing techniques target specific low-level features, such as color, shape and texture, showcasing good adaptability to the highly variable nature of trademark imagery [97]. Boia et al. proposed a CBIR system for recognition of logos [98], invariant to scale changes, color variations and lighting. The methodology followed by the authors is based on the bag-of-words technique and the SIFT algorithm, applied to small datasets with an evaluation limited by the number of images and different classes. A few years later, Jabeen *et al*. presented a CBIR system where they merge the features obtained by the SURF algorithm and the retinal fast key point feature descriptors (FREAK) [99]. Despite reducing the semantic gap between high- and low-level features, the method presents the highest accuracy value of 86% for the Corel 1k dataset, as well as not providing any color information.

### Machine learning approaches

The latest trend in modernizing information retrieval systems comes in the form of hybridizing such implementations with a range of machine learning techniques. Deep Learning techniques, in particular, have seen massive success due to the particularity of artificial networks to perform representation learning, which is useful in a wide range of retrieval tasks applied to images.

The earliest mentions of Deep Learning being successfully deployed in image-based scenarios originate from around the same time when Convolutional Neural Networks started gaining traction as researchers were made aware of their feature extraction capabilities. In 2012, Krizhevsky *et al*. [100] proposed the AlexNet architecture for image classification problems, which achieved state-of-the-art accuracy in several competition datasets, and motivated several efforts to improve the performance of image retrieval tasks in the following years. In the same year, Kang *et al*. introduced a multi-view approach to feature hashing, by training individual layers of the network with different views of the data, achieving a degree of scale invariance and learning shared features for multiple representations of a given object [101]. Later, Zhang *et al*. proposed a supervised methodology for simultaneous training of image features and optimization of hash functions [102].

Around 2015, a significant portion of published works relied on the computation capabilities of convolutional neural networks, resulting in a halt in the advancement of the cutting-edge technology due to the perceived ceiling of discriminative capacity being reached. As such, after that period, there has been an accentuated growth in research targeted at improving inter-class separation and intra-class condensation (i.e.: discriminative power) of features extracted with convolutional neural networks. In 2016, Gordo *et al*. proposed Deep Image Retrieval (DIR), a deep-learning technique that introduces a triplet loss function to improve similarity and dissimilarity computations between image embeddings [103]. Varga *et al*. outline a supervised method for concurrent training of semantic and CNN image features through hash functions, resulting in improved retrieval times and reduced computational load for the underlying system [104]. Similarly, Tzelepi *et al*. present an alternative feature extraction framework by using the information retrieved at the convolutional base of the network, rather than the convolutional layers on top, vastly improving extraction times and, in some cases, accuracy, in exchange for slower retrieval times, given the enlarged feature vector dimensionality [105]. Singh *et al*. propose a similar CNN-Based feature extractor for celebrity facial image retrieval, prioritizing the usage of convolutional layer outputs and max-pooling operations for dimensionality reduction in representing the repeated patterns in facial images [106]. Sezavar *et al*. proposed an image similarity search methodology in which CNN features are sparsely represented to reduce computational costs and thus significantly improving retrieval times at the cost of state-of-the-art precision [107]. However, in cases that a sparse representation is not possible, the retrieval time increases substantially. In 2018, Radenović *et al*. proposed a replacement for the typical similarity ranking metrics, introducing Deep Relevance Ranking (DRR), a method by which the learned representations of images are ordered by semantic relevance, achieving high-performance retrieval results that are more in line with human perception [108]. More recently, in 2023, Öztürk *et al*. introduced a novel method for content-based image retrieval (CBIR) in medical image datasets, leveraging OCAM (Optimized Contrastive Adaptive Margin), a triplet learning function with an improved objective that considers distances between positive and negative classes and an adaptive margin value. The study demonstrates the effectiveness of OCAM in improving CBIR performance on various medical image datasets, outperforming state-of-the-art approaches. The paper highlights the potential for further improvement in CBIR tasks through advanced loss functions and architectural modifications [20].

A niche area of research surrounding the hybridization of deep neural networks has also gained some traction in recent years. Convolutional Siamese Neural Networks (CSNN), introduced by Zhang et al. as a robust solution to classify respiratory diseases from CT imagery [109]. CSNNs work by utilizing stationary weights during simultaneous inference on two distinct input vectors, performing direct comparison between the two. Zhu et al. proposed Deep Generative Query Networks (DGQN) to improve image retrieval flexibility and quality by generating variations of ground-truth image results, which are then computed and retrieved by traditional CNN-based methods [110]. The proposed method has important limitations such as the low quality of the results produced and the restriction on the type of data to which it can be applied. Self-supervision mechanisms also serve a purpose in situations where the retrieval dataset is partially or completely unlabeled, which is a common occurrence for trademark image data. Chen *et al*. propose a self-supervising technique based on the averaging of embedding information extracted from multiple augmentations of a given image, encouraging researchers to explore self-supervising techniques as a highly performant alternative to otherwise fully supervised or unsupervised machine learning [111]. More recently, Monowar *et al*. propose AutoRet, a self-supervised DCNN that extracts and combines feature vectors from multiple patches of image data [49].

The most recent motivation for CBIR appears to be cloud computing technologies, where most of the burden that comes with the processing of massive amounts of information is outsourced to specialized computing units, offering some degree of democratization of high-end computing resources. Anju & Shreelekshmi propose a highly scalable image retrieval system based on cloud-based image clustering and indexing, offering state-of-the-art retrieval performance on the Corel-1k and Corel-10k datasets [112]. Wentao *et al*. present a cloud-based DCNN feature extractor fine-tuned for encrypted image processing, ensuring top-tier performance, and vastly improved privacy preservation and computational costs due to the image encryption phase [113].

When focusing on image retrieval tasks regarding Industrial Property, some works stand out as key contributors to the current state-of-the-art in this matter. Wang *et al*. propose a trademark image retrieval system based on the pairing between a CNN-Based discriminative feature extractor and a retrieval algorithm based on a simple similarity matching algorithm [114]. Perez *et al*. propose a twice-trained CNN approach, where one of the networks was trained to recognize perceived similarity, and the other was trained to detect conceptual similarity, having achieved unparalleled accuracy on the METU trademark dataset [37]. More recently, Trappey & Trappey propose an adaptation of the triplet-loss learning mechanism for trademark image datasets, where each training sample is comprised of an anchor image, a dissimilar example and a similar example, greatly improving retrieval accuracy in the Logo2K dataset [6]. Cao *et al*. propose an unsupervised trademark image retrieval system based on attention mechanisms, introducing bias towards relevant features during training. Their system matches or surpasses traditional feature extraction methods and even some supervised systems on the METU trademark dataset [115]. These last two proposals have their performance evaluation limited to datasets restricted in number of images, as well as their content variability. Jardim *et al*. propose a multiple feature extraction workflow for trademark image retrieval, by leveraging the extractive potential of convolutional layers. Information from several views of the same input image is combined to achieve deeper levels of representation and obtain superior retrieval performance in unlabeled datasets [116].

## 3 Methodology

DarwinGSE, short for Darwin Graphical Search Engine, is an advanced search engine that was developed as a contribution to an underdeveloped market (Intellectual Property Sector). It is a trademark image retrieval system built to overcome the difficulties raised previously. The proposed system was constructed from scratch to be specifically effective in Intellectual Property problems, and contains design choices that are unique to match the requirements in this industry. This section begins by providing a general description of the pretended system functionality, and develops further into each of the modules used.

### System description

To begin interacting with the system, the user is confronted by a simple interface in which he can provide a query, typically an image upload, and initialize a search request. As soon as the content is processed, the user can then decide to refine his search via the several configuration options provided. When they are satisfied, the results are computed and presented, allowing even more interactivity in the form of selecting potentially conflicting trademarks to be included in an automatically generated report. While the standard usage loop stems from the method by which images are compared and sorted, additional systems are constantly working in the background to ensure that a) the provided data is up to date, and b) the search parameters are adequately matched to the query type. The symbiotic nature of the components we use is, to our knowledge, a new, interesting contribution to the literature regarding this industry, hence why in the following sections, we will contextualize the functioning methods behind three sizeable modules of DarwinGSE.

### System components

**Fig 3:**
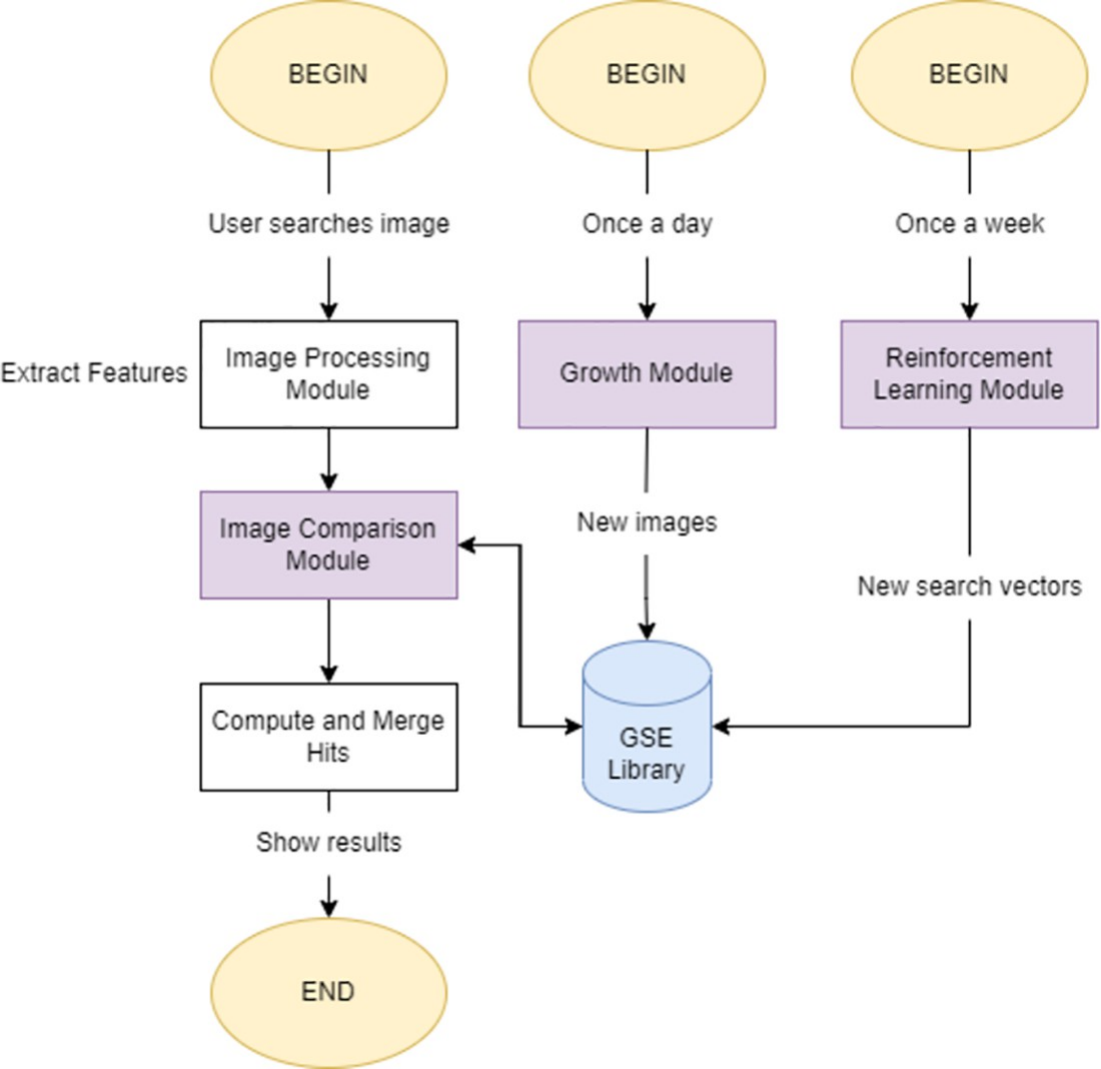
Minimal representation of the DarwinGSE system.

As a whole, DarwinGSE is comprised of several individual modules that work in a shared environment and carry out different, though equally important, tasks that allow for a high degree of confidence in the results we present. As a starting point, refer to Fig 3 for a minimal representation of the proposed system. The purple-colored blocks refer to the three main modules we will detail in this paper, however, some mentions to the other processes are also present throughout the chapters.

The proposed approach follows a simple architecture based on several possible triggers for each of the events. Through a web interface, the user can submit an image of any supported format, and configure a series of filters to customize the depth of the search. After real-time feature extraction, the **image comparison module** confronts all relevant pairs through the combination of multiple features. Simultaneously, this module includes a global FAISS index search algorithm that looks for high similarity hits, focusing on text and conceptual contents.

On a frequent basis, the **growth** and **reinforcement learning** modules ensure that the knowledge base remains up-to-date, and that results are as accurate as possible. Facing the number of trademark registrations that happen every week, new trademark images are processed every day for features, and the data libraries that back the searches are updated with the least downtime possible. Every week, the data collected by the relevance feedback system is analyzed and modelled. According to the quality of the recent results, new search hyper-parameters are generated, extending or reducing the lenience of the system, and applying modified combinations of features to improve results over time.

## 4 Image comparison module

The comparison module is the backbone of every search request our system receives. Its base functionality is providing on-demand graphical searches, but as new challenges and requirements developed, we have since developed user-operated filtering tools and text searching capabilities. In its current version, an advanced catalog of IP vigilance tools is provided in a simple to use, straightforward package.

### Composition

**Fig 4:**
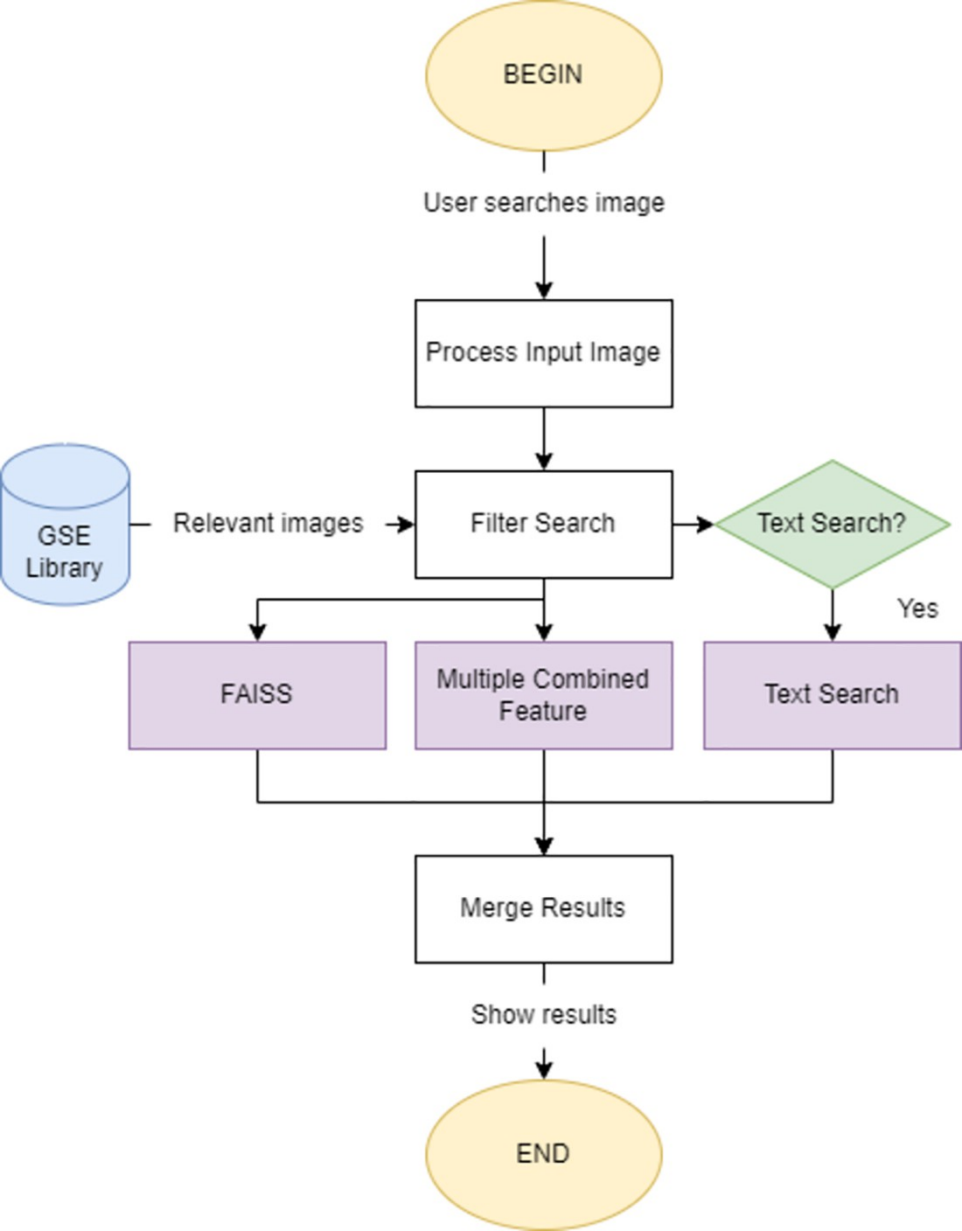
Simplified diagram of the image comparison module.

When a user submits an image to the system, it is processed in real-time with a combination of feature descriptors, resulting in two distinct layers by which results are computed. The base of every comparison is the combination of Combined Feature Searches and Facebook AI Similarity Searches (FAISS), which vary in terms of the evaluated image aspects, but are ultimately merged into a single, unified result set. Additionally, a pair of optional arguments can be defined to extend searches to textual data, or provide some control to the depth of the search, regarding IP-specific Nice Classes and Jurisdictions. See Fig 4 for a simplified representation of the comparison module, and consult section *Image processing* for more details on how images are processed.

### Operation

Out of all the modules detailed in this paper, the comparison system we use in DarwinGSE is the most restrictive due to its very high efficiency demands, after all, if a graphical search engine does not produce results in a timely fashion, it becomes frustrating to use. This is further aggravated given the large dimensions of the dataset, and the complexity of the algorithms required to extract meaningful information from unlabeled images. In the following subsections, we introduce the techniques we developed to achieve real-time performance with state-of-the-art result quality.

#### Multiple combined features

**Fig 5:**
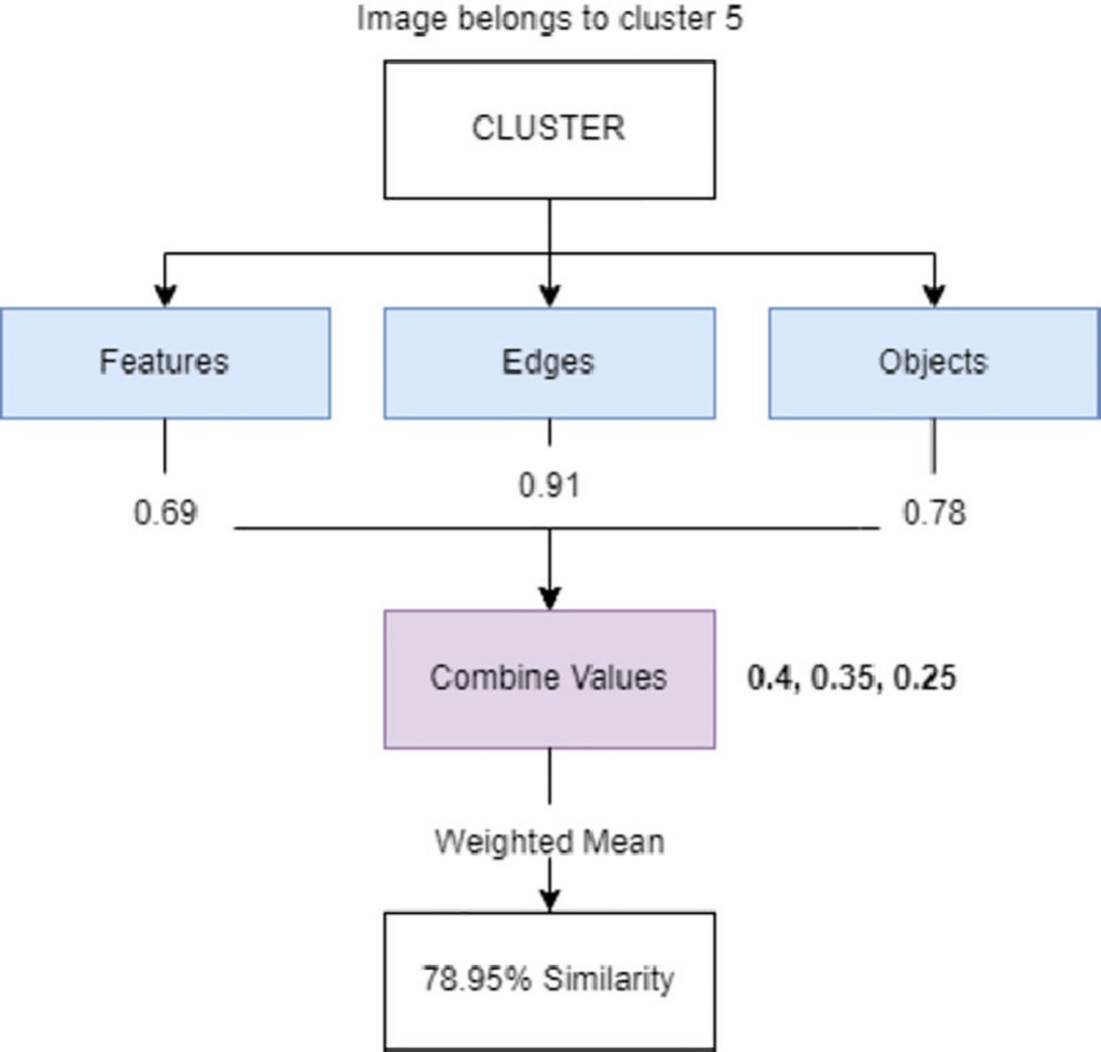
The system combines all three similarity scores into a single one.

In traditional approaches, the confrontation of two images’ feature vectors is enough to provide a good similarity score between them, since the evaluators (models) used to extract these features are usually trained to know what to look for in datasets of lower dimensions. When dealing with trademark images, generalization is the key, hence why we opted for the extraction of multiple feature vectors, weighed separately and combined to represent a single similarity score between 0% and 100%. Currently, we evaluate images with respect to their general features (original figure data), edge-wise features (a *Canny Edge* representation is used), and objects (we use a proprietary system based on *Watershedding K-Means segments*). To assign a degree of importance to each characteristic, we use a **Weight Vector** representing the mass of each component in the final formula. Through this method, not only are we introducing additional information to the comparing methods, which in turn may improve result quality, but also enabling the customization the individual **weight values** for each cluster, depending on its content. For instance, if we understand that a certain cluster contains mostly black and white images, we may opt to bring the edge-wise feature weight higher to the detriment of the remaining features. A visual representation of the multiple combined feature formula operation can be seen in Fig 5. This approach to feature evaluation is supported by one of our previously published works [117].

Furthermore, the final multiple combined feature similarity score between two images, *A* and *B*, is given by Eq (1), where:

- *A* and *B* are three-position lists containing vector representations of the image features (*n* = 1), edges (*n* = 2) and objects (*n* = 3).
- *W* is a three-position vector containing the weights of each component. The sum of all values in *W* must equal to 1.
- No values in Eq 1 are inferior to 0.
- The result of the equation is no greater than 1, and no lower than 0.

$$\begin{array}{c} Sim_{AB}=\sum_{n=1}^{3}\left(\left(1-\frac{A_{n}\cdot B_{n}}{\left|\right|A_{n}\left|\right|\times \left|\right|B_{n}\left|\right|}\right)\times W_{n}\right) \end{array}$$ (1)

#### Cluster-specific searches

**Fig 6:**
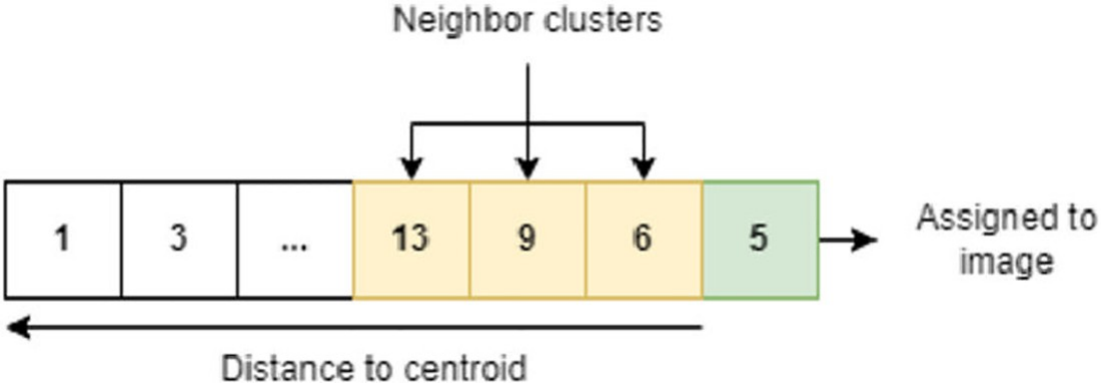
Cluster extension mechanism. Green: the submitted image’s cluster. Yellow: Extended cluster range containing close-by centroids. White: remaining, far centroids.

To improve performance and avoid unnecessary resource consumption, we implemented a cluster specific approach to image storage and comparison. The rationale for this method is that certain images are predisposed to represent higher similarity values with any given input, while others are so distinct in content that comparing them is not required to provide high-quality results. As such, we initialized *K* = 21 clusters (through a silhouette method) of low within variation and high outside variation (similar images in a cluster, dissimilar content between clusters). Initially, when a certain image was submitted into the system, it would only be compared with other images belonging in the same cluster. Upon further testing, we noticed that graphical search result quality improved significantly when clusters within *d* = 0.15 Euclidean distance were also considered, meaning that the image is compared with all neighboring clusters (see Fig 6).

#### FAISS index

Alongside the typical feature vectors, DarwinGSE stands out from typical retrieval systems due to its basic ability to conceptually match images with similar semantic meanings. We achieved a degree of visual understanding by extracting embeddings from every image with a Sentence Transformer model. Sentence Transformers are high-depth machine learning models capable of extracting image or text embeddings for accurate semantic similarity evaluation. One type of Sentence Transformers leverages Contrastive Language-Image Pre-Training (CLIP) to produce image-text embeddings in a single, shared coordinate space, making it extremely useful for text-to-image or image-to-image searching [118]. As part of our processing mechanism, we extract 512-dimension embeddings with a CLIP Sentence Transformer (CLIP-ViT-B-32), however, the resulting vectors are simply far too large for traditional pairwise comparison, which is why we opted for Facebook AI Similarity Search (FAISS) indexes to store and search our CLIP embeddings. FAISS is a library developed by Meta for efficient clustering of dense vectors, allowing for massive datasets (bigger than available RAM) to be searched in a very short amount of time. It works with its own data type, an *index* that stores vectors of any size, and provides simple L2 or dot product search functions for exact or approximate searching. When compared to a vectorized pairwise comparison of over 4 million vectors, FAISS indexing is faster to search and lighter to store (see Table 1).

**Table 1:** Traditional VS FAISS searching.

Metric	Vectorized Pairwise Comparison	FAISS
Library File Size	9GB	1.1GB
File Load Time	15s	1.5s
Search Time	14s	0.9s

One drawback of this implementation is that FAISS index performance is highly dependent on appropriate hyper parametrization, and requires long training times to achieve the desired performance. Considering the ever-expanding nature of the IP dataset DarwinGSE is built upon, we felt the need to employ automated techniques to automate the construction of these indexes on a daily basis. Regarding how we achieved this, refer to section *FAISS updater* of the document.

#### Extended options

This section refers to the additional functions aimed at improving the variety of search scenarios supported by the system. More specifically, we refer to the settings the user can adjust to increase or decrease search depth, as well as text-based functions that cover an even wider range of image types found in our dataset.

*Filtering*. When classifying trademark images, there are two main ways to segment data: Nice classes and Countries of Jurisdiction. *Nice Classification* is an internationally established system for categorizing trademarks into 34 goods and 11 services. While not directly assigned to images, the underlying trademark information must contain one or more Nice classes, meaning we can create data filters for however many classes the user may want to search on. To make multi-class filtering possible, the dataset libraries contain information about each record’s Nice classes. When a filtered search is requested, Regex filters are applied during the data loading process, meaning the subsequent processes only work with clean data, avoiding unnecessary computations on unwanted records. Furthermore, every record in our knowledge base pertains to a trademark registration originating in a given country or alliance (i.e. European Union). With this, we define our second filtering option, since a user may not wish to see results from a country that does not overlap with their trademark. To accommodate filters by country, we perform *ISIN* filtering with the Pandas library.

**Fig 7:**
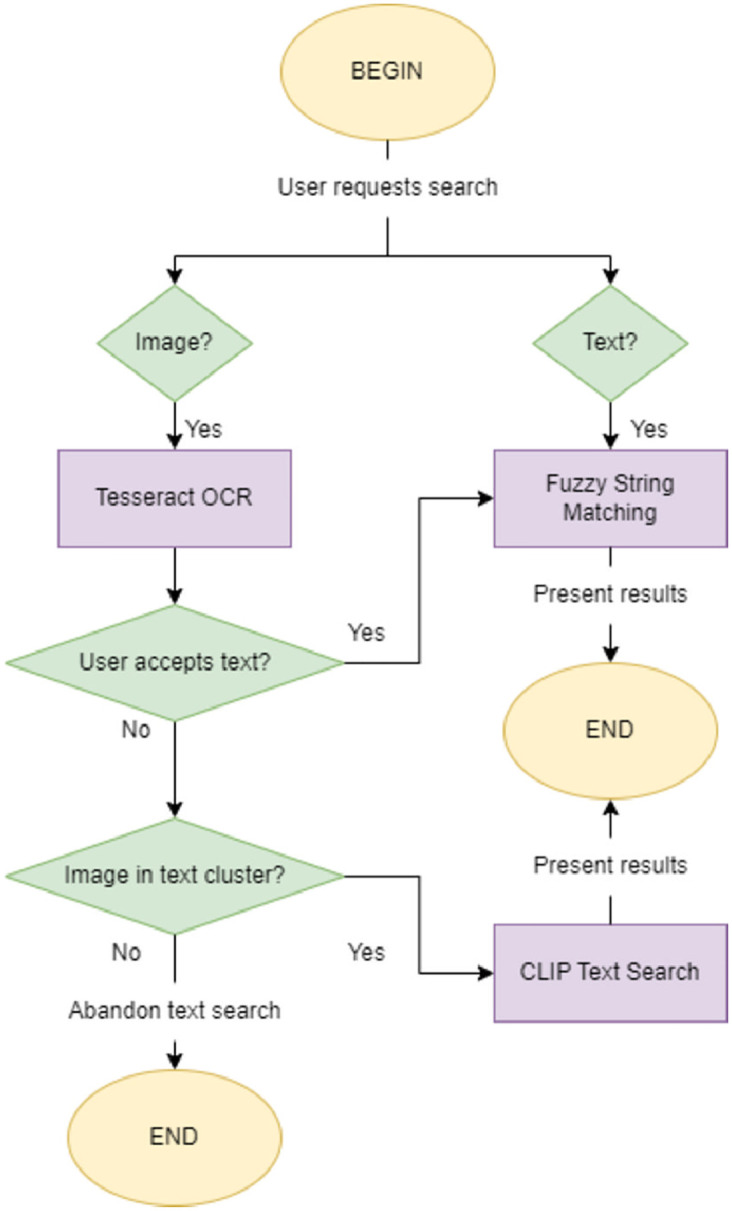
Text-Based search suggestion workflow.

*Text searches*. In general, searched images can be predominantly graphical, textual, or a hybrid of the two (graphism with text components). The previous comparison mechanisms do a great job at managing graphical and hybrid requests but under-perform when the submitted image is based on text only. Text-only searches were initially considered edge-case scenarios, however, upon further investigation, we estimate that a respectable 19% of our dataset is comprised of images that are comprised of only text components, making text-wise comparison something to consider. Text-based searches were implemented in a variety of ways: (a) the user can submit text directly; (b) the submitted image is scanned for text, using an OCR algorithm, or (c) the submitted image belongs to a cluster where most images are text-based. In the first two cases, a Fuzzy String matching algorithm is used for computing the text-based result set, whereas in the remaining option, we present the FAISS result set as the text result set, since the extracted embeddings are excellent for describing text features. A simple representation of the text search switch-case system is presented in Fig 7. We found that the implementation of this subsystem has significantly improved search quality in edge-case scenarios, and consider it a valuable addition to any IP protection system.

## 5 Reinforcement learning module

Given the complexity and multi-parameter nature of the image comparison module, manually optimizing the underlying weight vectors and threshold values is impractical. In the sense of system modernization by way of machine learning applications, we developed a proprietary module that automates this task, while also being completely transparent to the user. We believe that maintaining an uninterrupted searching experience (no in-page forms, questions or other intrusive feedback modules), while still generating useful information for continued improvement is mutually beneficial and ideal.

### Composition

**Fig 8:**
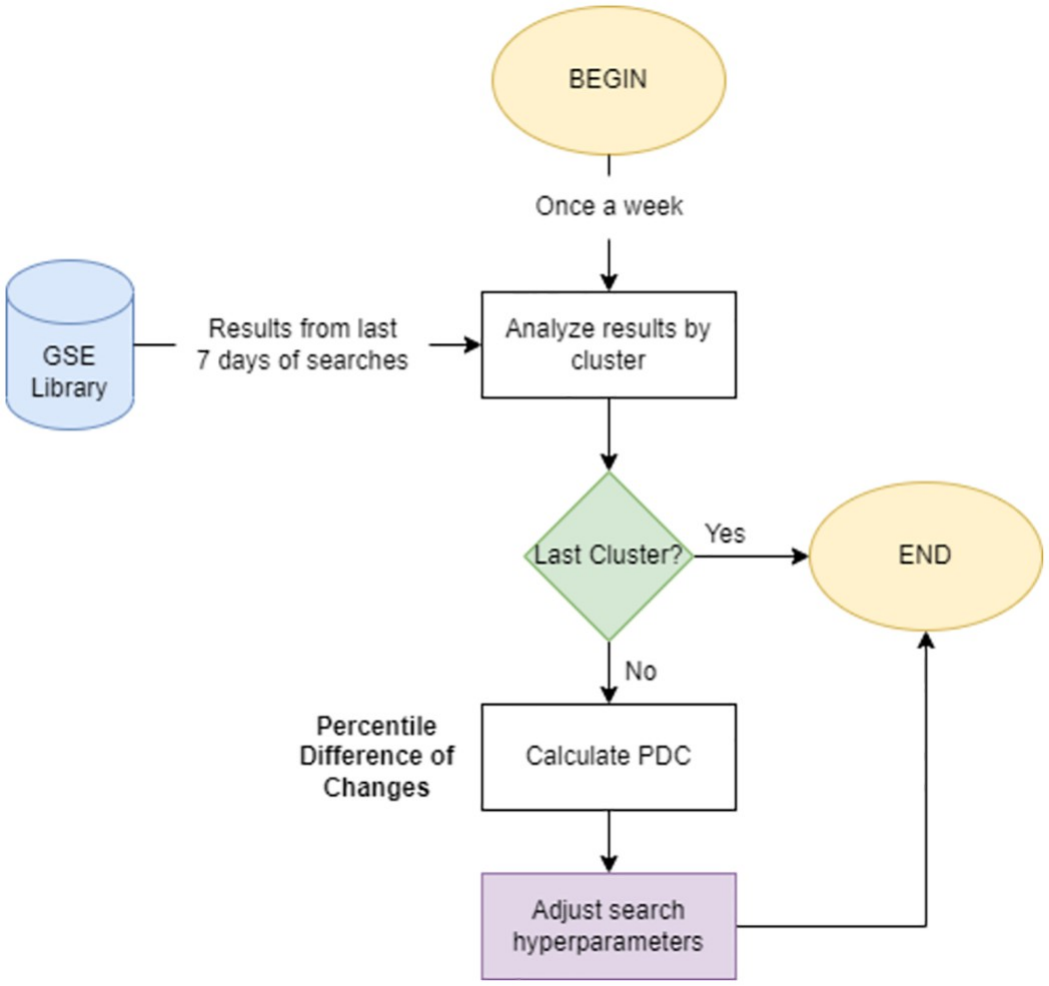
Simplified diagram for the reinforcement learning module.

This module works by the principle that constant and unbiased usage of the system will, over time, provide enough feedback data to reveal the optimal hyper-parameters for the best possible search results. See Fig 8 for a simplified diagram of the reinforcement learning module.

The core of this algorithm is a hybrid **Reinforcement Learning** method, a branch of machine learning training mechanisms that introduces a reward system for desirable outcomes and punishes undesirable results, working on a trial-and-error basis.

### Operation

While this module has some resemblance to traditional machine learning applications, there are some distinguishing factors that further solidify its novelty. The following subsections aim to provide some detailed information on how the module works and how the system design choices were leveraged to produce an effective optimization system.

#### Transparency

**Fig 9:**
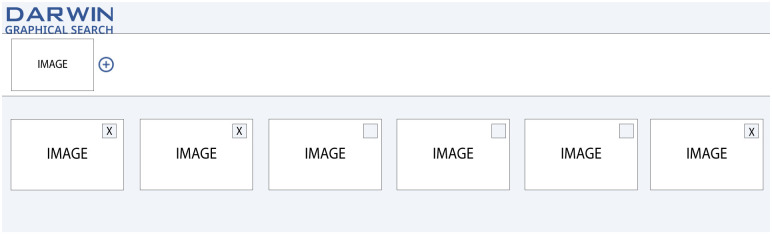
The DarwinGSE result page is straightforward and easy to use. Reproduced under a CC BY license, with permission from Techframe—Sistemas de Informação, SA, original copyright 2023.

As previously mentioned, the machine learning mechanisms in place have the particularity of recovering the necessary training data in a manner that is 100% transparent to the user. As a result of UX studies performed by both front-end and back-end engineers, we created a design that is both easy to use and contains enough interactivity to generate useful insights on any result set’s quality. We provide a simplified view of the result page in Fig 9.

In this screen, the user can freely add and remove images to a selection that will be used to generate a custom report with the respective trademark’s relevant information. On the result page, any hit that is deemed as potentially conflicting (very high similarity score) is automatically pre-selected. Still, users can select/deselected images from the result set, according to their perception of what hits are adequate, and any changes to the selected hits are saved. At regular intervals, this user generated data is analyzed to identify patterns that represent the quality of recent searches. In this manner, we avoid the usage of intrusive feedback forms and questionnaires, allowing the uninterrupted filtering of user results to generate the data we need.

#### Percentile difference of changes

When studying ways to leverage the collected data, we felt the need to develop meaningful metrics that could be easily interpreted and modeled into several potential outcomes. As such, we defined the **Percentile Difference of Changes** (PDC), a set of three independent metrics that represent key takeaways from how a user performed their selection. They are:

- *Percentage of changes (PC)*—A percent value representing whether the number of added/removed images is superior or inferior to the original pre-selection. We can also invert this percentage to estimate the accuracy of the pre-selection system
- *Distance of changes (DC)*—Whether the observed changes were performed on the upper or lower range of the result set, which may represent an incorrect threshold value for the pre-selection system.
- *Amplitude of changes (AC)*—Whether the similarity percentage of the added/removed images is high or low, regardless of its position in the result set. Adding a low similarity result to the selection can signify a steep decline in the quality of a result set.

These metrics are the backbone of the reinforcement learning module, and determine the breadth of adjustment necessary for any given set of parameters. Naturally, we implemented a decision support system to aid in making these choices.

#### Decision tree

With the limited amount of information that the **PDC** metrics provide, we strived to keep the decision support system simple, easily manageable, and fast. For this, we opted for a shallow decision tree (*D* = 5) that allows us to efficiently map the following condition cases:

- *PC* > 25%—An effective accuracy of below 75% means the user required a considerable number of changes to achieve an adequate result set. This instantiates a new weight vector with a variation of 15%, meaning each of the three components can shift either way by 0.15, while still adding up to a 1.00 total distributed weight.
- *DC* < 15% *and AC* < 0.21%—Good accuracy and small distance metrics represent an adequate result set, with most differences being attributed to incorrect threshold values for the preselection system. The threshold values are updated according to *DC*, with higher distances requiring more leniency (lower thresholds).
- 15% < *DC* < 60% *and* 0.20% < *AC* < 70%—If the change amplitudes fall between this range, the preselection system produced adequate selections on an otherwise inadequate result set. While not as extreme as the first condition, these values instantiate a new weight vector with a low variation of up to 3%.
- *DC* > 60% and *AC* > 0.70%—Essentially represents an aggravation of the previous condition, with a lower quality result set. The new vector, in this case, is instanced with a variation of up to 6%.
- *PC* < 8%—Represents an effective accuracy of above 92%, which is the value defined for good performance on both the preselection system (thresholds) and the underlying weight vector. No changes are applied at this point.

**Fig 10:**
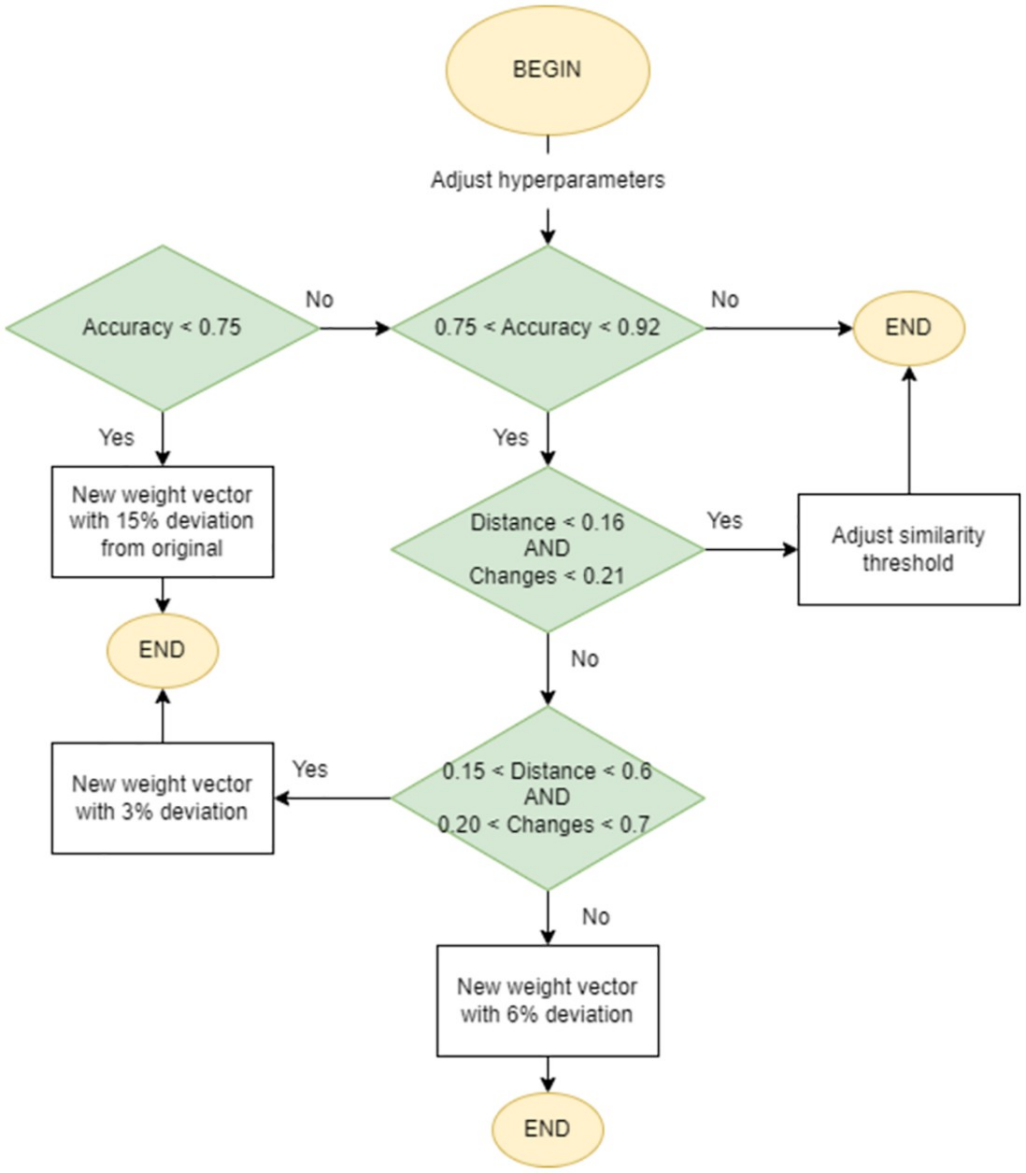
Decision tree for the reinforcement learning mechanism.

For better visualization of all case scenarios, a comprehensive graph was developed and can be consulted in Fig 10.

#### Optimization frequency

Setting an appropriate recurrence period for the adjustments to be made is an important task. On one end of the spectrum, optimizing too often would mean a given weight vector might not get enough time to be properly tested across different search scenarios. On the other, having too much time in between optimization cycles can negatively impact user experience, especially when an inadequate vector is in use for extended periods of time. To counter this, we studied internally generated usage data (from simulated testing across 72 days) to find a balanced time frame for the learning mechanisms to take place. As such, the ideal frequency should include searches on as many of the 21 clusters as possible, while avoiding diminishing returns from extended usage of potentially incorrect hyper-parameters. We tested *N* = [1, 3, 7, 14] for the number of days between each learning epoch, relative to the average number of searches, clusters reached, and total evaluated results for each recurrence period. Note that the number of evaluated results does not equal to the total number of results, but rather the portion of those results that have been considered by the user (i.e. if the user did not interact with the second page of results, we consider only the first page as the total evaluated results). Our findings can be consulted in Table 2.

**Table 2:** User interaction and search depth for recurrence period, in days.

N	Searches	Clusters*	Total Evaluated Results
1	56	4	2360
3	136	7	5680
7	310	15	13920
14	678	16	27360

*Maximum of 21 clusters. *N* = [1, 3, 7, 14].

We see an expected steady growth of total evaluated results as the recurrence period increases, which in turn signifies a larger amount of training data. However, we notice that between *N* = 7 and *N* = 14, only one more cluster has been reached, on average, per twice the time. This means that some image clusters are more often searched than others, and thus having larger recurrence periods does not translate into more cluster coverage per instance of optimization. We settled on *N* = 7 for the optimization frequency of our reinforcement learning module, covering an average of 71% of clusters with 14000 relevant training rows per cycle.

## 6 Growth module

Often, image retrieval systems are trained for extended periods of time on moderately sized datasets, and tend to achieve significant retrieval accuracy given that the data stays relatively consistent in terms of its content. IP datasets have the particularity of being highly heterogeneous, since trademark images are not confined to specific classes, patterns, and complexity. Moreover, with the number of trademark registrations submitted every day, any system built to work with intellectual property datasets needs to be updated very frequently, as the lack of up-to-date results can lead to IP theft, copyright issues, and financial loss. For DarwinGSE, data recency is a critical factor and several algorithms have been developed to act as quickly as possible in making all new data available in a timely fashion.

### Composition

**Fig 11:**
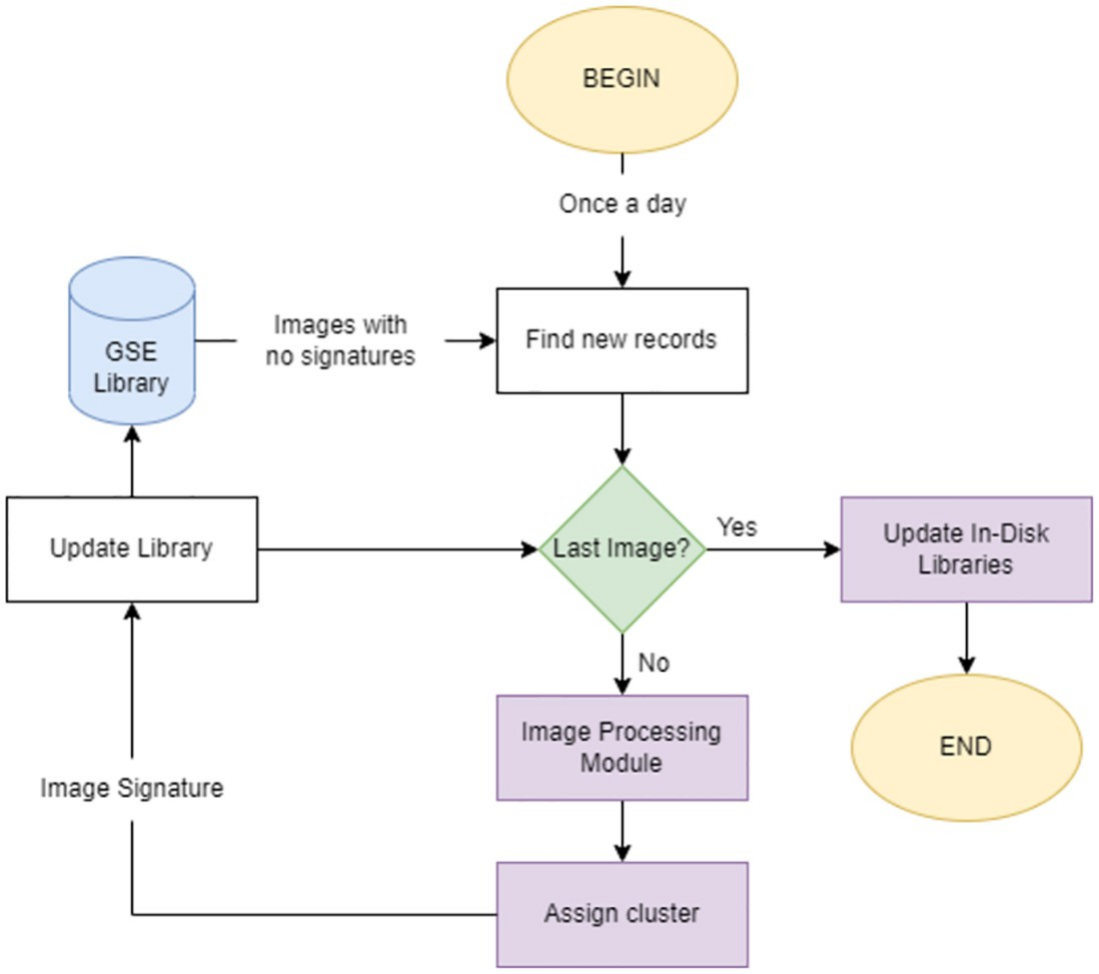
Simplified diagram for the growth module.

There are multiple individual components that all act in unison to update our knowledge bases. On average, our back-end services receive 2500 files per day, requiring a relatively short processing time to ensure no backlog is formed as time progresses. We developed a recurrent algorithm that scans the graphical knowledge base for unprocessed records, and creates a list of image paths that are subsequently processed in accordance to our preset image processing module. Every image is then assigned to one of the 21 preexisting image clusters. Finally, we collect all processed records and write new in-disk libraries, keeping the system up to date without any downtime on the search functions. The diagram for the growth module can be consulted in Fig 11.

### Operation

Below, we detail the individual subsystems that allow for efficient image processing and, ultimately, for our graphical searches to extend accordingly. This module has been evaluated to process up to 47000 images per day, far surpassing the estimated daily inflow of 2500.

#### Image processing

Most of the time spent updating the database is used in individually processing every new record (image). In this step, all records are processed with the same morphological methods as any user-submitted input. First, the image is normalized to 224 × 224 resolution, fitting the input layer of the Deep Convolutional Neural Network (DCNN) adopted for the task (variation of the VGG16 network). Several augmentation and quality improvement techniques are employed to reduce noise, color over-complexity, region overlapping, and bad cropping, before an edge-representation of the image is generated and its relevant regions extracted, completing the three-component image description used as the basis for every comparison. Finally, we use a sentence transformer to extract a 512-position sentence embedding of a given image. Due to the information they carry, embeddings are treated differently during graphical searches and require vastly different structures to work in the best way possible, both of which are detailed in the following subsections about in-disk libraries and indexing. The developed method is described in richer detail and supported by one of our previously published works [116].

#### Assigning clusters

When assigning clusters to new images, we found that using a Euclidean Distance based approach provided accurate and timely classification. A given image’s feature vector is confronted with all 21 centroid coordinates, and distances are ordered ascendingly. We take the nearest cluster’s label and assign it to the image. When compared to a K-Nearest Neighbors approach, we conclude that the improved classification speed far outweighs any measurable improvement in accuracy. Daily, after processing all new records, we generate 21 files, each one containing all image data for one cluster. This in-disk approach enables I/O dependent data loading, as opposed to SQL to Python layers that cause significant overhead when dealing with large amounts of data.

#### In-disk libraries

DarwinGSE’s graphical searches work on top of a complex SQL database of image signatures. This structure brings many ease-of-use advantages, accepting the storage of python specific data formats with minimal conversion, but ultimately requiring communication to the graphical search API for every request. To improve future-proofness, we studied the theoretical time consumption of the data transfer process between Python and SQL (see Table 3), and found that with the expected growth of search depth, transferring data between platforms would quickly become unviable for a real-time system.

**Table 3:** SQL to Python data transfer overhead analysis.

Number of Rows	Query Time (seconds)	Transfer Overhead (seconds)
150000	1.20	2.21
300000	1.89	9.76
700000	3.52	14.80
1500000	5.01	45.42

To solve this issue, we opted for localizing the knowledge base in a series of 21 highly optimized files specifically designed to hold *Pandas Dataframe* information, *Feather-Format*. Essentially, this removes the need to convert data formats and turns cluster file reading into an I/O dependent task, rather than a server-client transfer. The consolidated time-to-read for equivalent row counts can be consulted in Table 4. Upon testing, we found *Feather-Format* provides more linear scaling between the number of rows, and the time needed for reading.

**Table 4:** Feather-Format file reading times.

Number of Rows	Read Time (seconds)
150000	1.257
300000	1.534
700000	2.840
1500000	6.568

#### FAISS updater

FAISS indexes are stationary structures, and their impressive search performance can be attributed, in part, to innate training capabilities that optimize inner product calculations depending on the contained data. In some cases, it is also possible to specify the creation of Voronoi Cells, structures similar to clusters in functionality and behavior, to improve search times even further. With this amount of adaptability and configuration, there is no one-fits-all setting that will accommodate our data as it grows, and manually adjusting hyper-parameters every time new images are added is impractical and counter intuitive. For this, we leverage *autofaiss*, an automatic parameter selection framework that optimizes index creation, given memory and latency constraints. This inverts the index creation workflow and enables us to set-and-forget constraints for the final index, such as the maximum allowed memory, the desired distance metric, and maximum search latency. All training specifications and further optimizations are automatically adjusted to fit within the specified constraints. To strike a fair balance between performance and result quality, we specified **4GB** of memory allowance, **2000ms** of search latency, and the approximate internal product distance metric as our index parameters. Naturally, these numbers are subject to punctual revision as both complexity and requirements evolve over time. As a general example, we provide all relevant FAISS index configuration values for an image knowledge base of size 4200000*x*512 (4.2 million vectors of size 512) in Table 5.

**Table 5:** FAISS index configuration and metrics.

Parameter/Metric	Value
Height	*h* = 2048
Voronoi Cells	12288
Probes	6144
Shape	(4207260, 512)^1^
Memory (Disk)	1.1GB
Similarity Metric	Internal Product
*Index Build Time*	136min
*Load Time*	1.5s
*Search Time*	909ms

^1^ Dataset shape according to a recent row count of trademarks available in DarwinGSE.

As evidenced by the table information, more than 2 hours per day are required to keep the FAISS index up to date with the best possible use-case performance. This is an extremely time-consuming task but ultimately sets the system apart by providing quick, semantically aware results to graphical searches.

## 7 Results

In this section, we will present a set of 5 (five) results achieved by using DarwinGSE with a variety of input types. To perform these searches, we use an internally developed web-based interface that communicates directly with a private API serving the graphical search module, therefore ensuring the reproducibility of the presented search results. While these results are meant to be visually interpreted by readers, we also share a short paragraph with our observations about each scenario. For each sample we present, we show the top-20 results, and unless otherwise specified, no additional filtering options were selected in any instance.

### Minimal complexity graphical search

**Fig 12:**
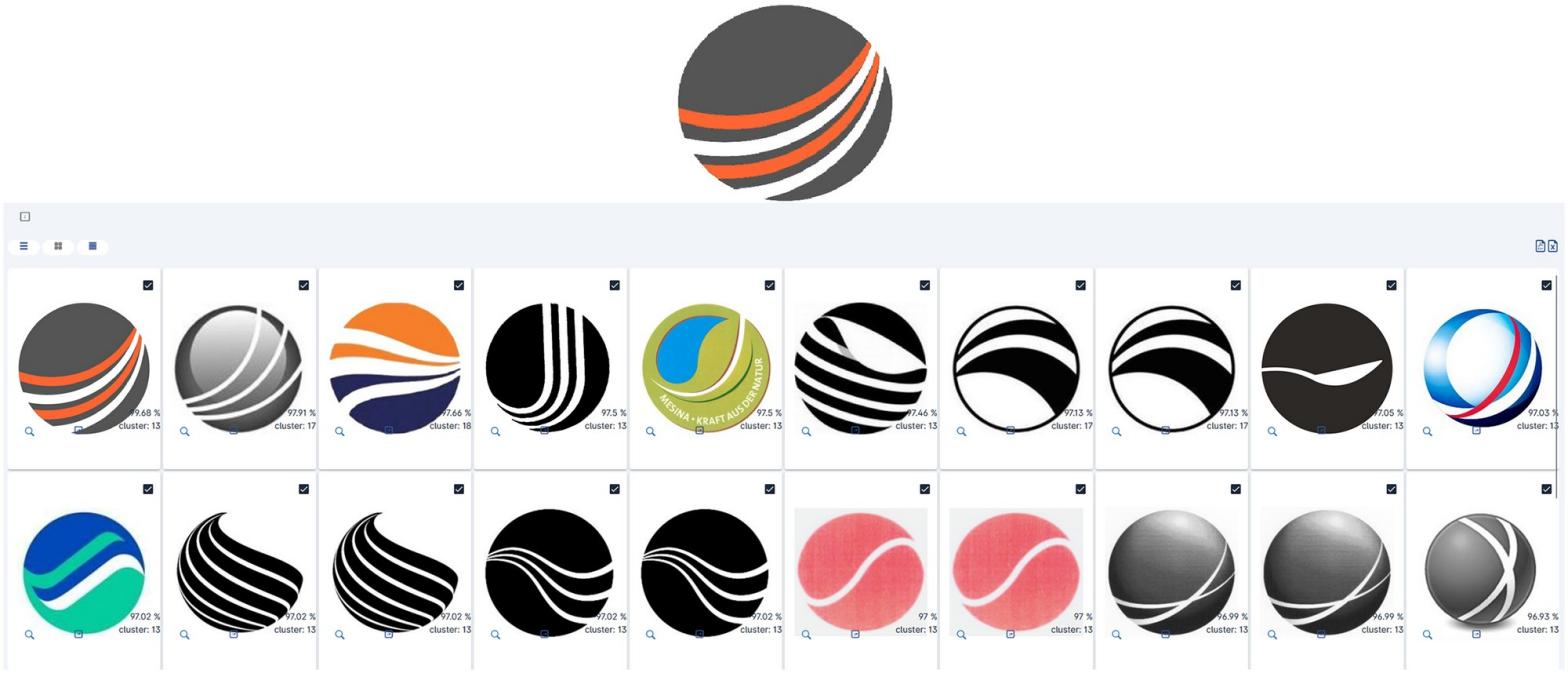
Sample A—Minimal complexity graphical search. Reproduced under a CC BY license, with permission from Techframe—Sistemas de Informação, SA, original copyright 2023.

The first result sample, shown in Fig 12, is meant to showcase the purely graphical capabilities of the system. We opted for an image with relatively low visual complexity, lacking text and focusing more on the illustrative side of trademark imagery. This sample aims to present the system’s functionality at its simplest level, establishing a baseline result. One key takeaway from this sample is the reflection of the input image’s texture on the result set, preserving the same curved line aesthetic across all results.

### High complexity graphical search

**Fig 13:**
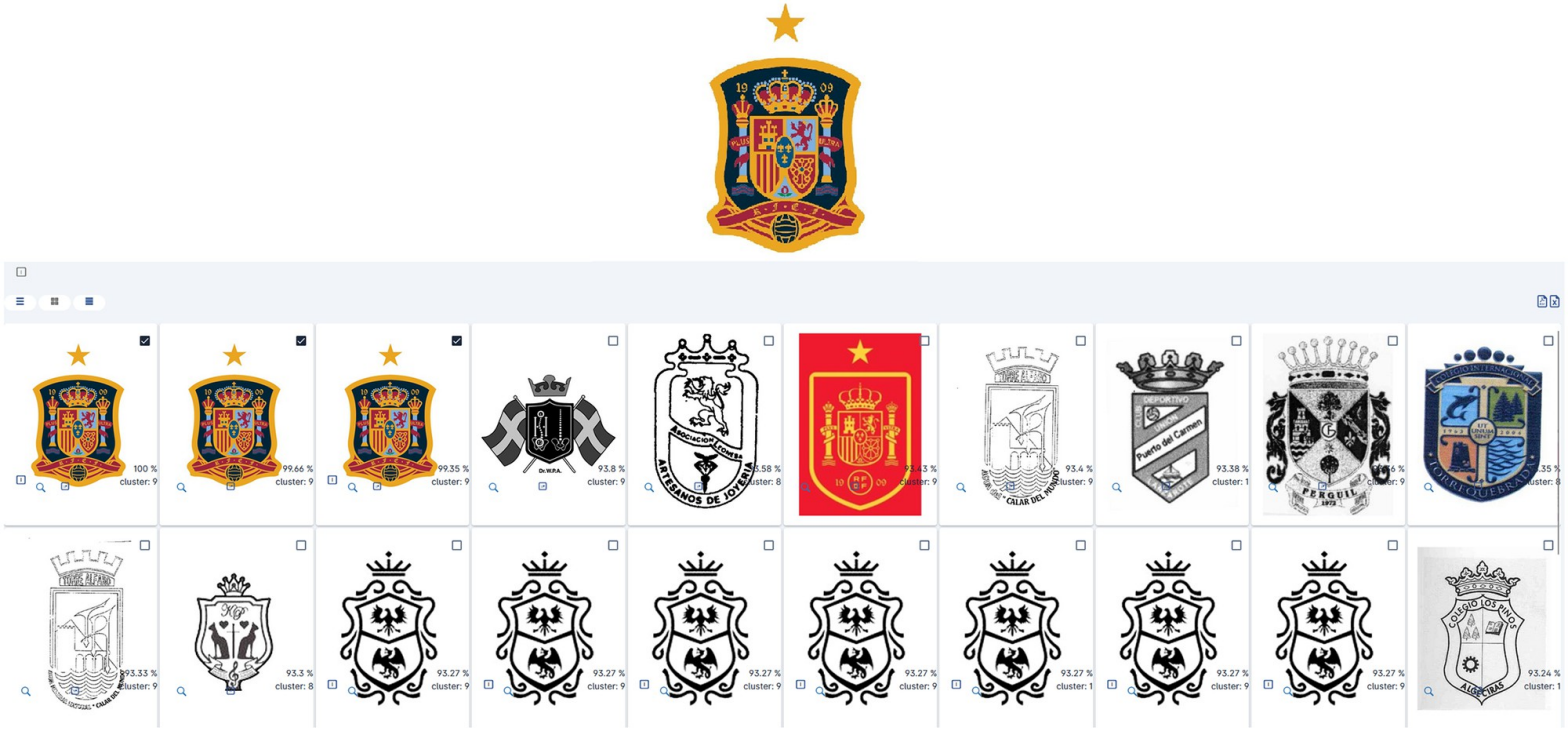
Sample A.2—High complexity graphical search. Reproduced under a CC BY license, with permission from Techframe—Sistemas de Informação, SA, original copyright 2023.

We further extend the graphical-only searches with a visually challenging example, like the one presented in Fig 13. We consider these container-type images to be typically difficult to process because they represent a larger concept through a very fine intertwining of smaller visual components (i.e. a banner or shield and all symbolism present within them). The system found some duplicates in the result set, but the overall representation of the input image was achieved.

### Graphical text search

**Fig 14:**
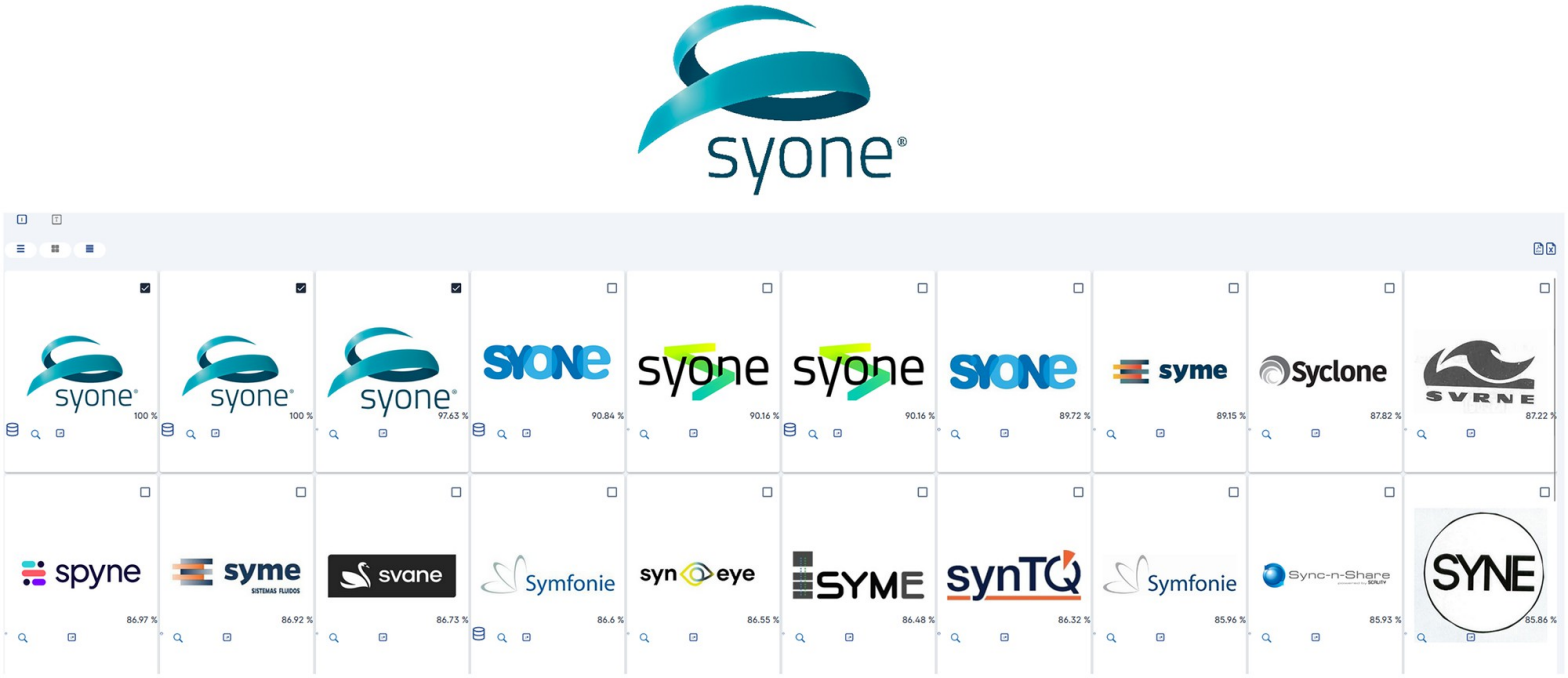
Sample B—Graphical text search. Reproduced under a CC BY license, with permission from Techframe—Sistemas de Informação, SA, original copyright 2023.

A major part of trademark images relies on text to broadcast the business name and create an association with their logo (Fig 14) hence justifying the importance of allowing textual components on a graphical search engine designed for trademark datasets. In these cases, we may use either of the three available text-aware components (user input, OCR, or sentence-encoders) to achieve similar results. Sentence-Encoding is fully automatic and requires no intervention by the user to produce textually aware searches, encoding all recognized text along with the image’s embeddings and thus relying on FAISS indexes to present similar results. In simpler instances, OCR may successfully extract such text from the image and suggest it to the user, who ultimately can decide whether to keep it or discard it. In case a higher level of control is required, a text box is also available to manually fill in and customize the text search payload.

### Text-only search

**Fig 15:**
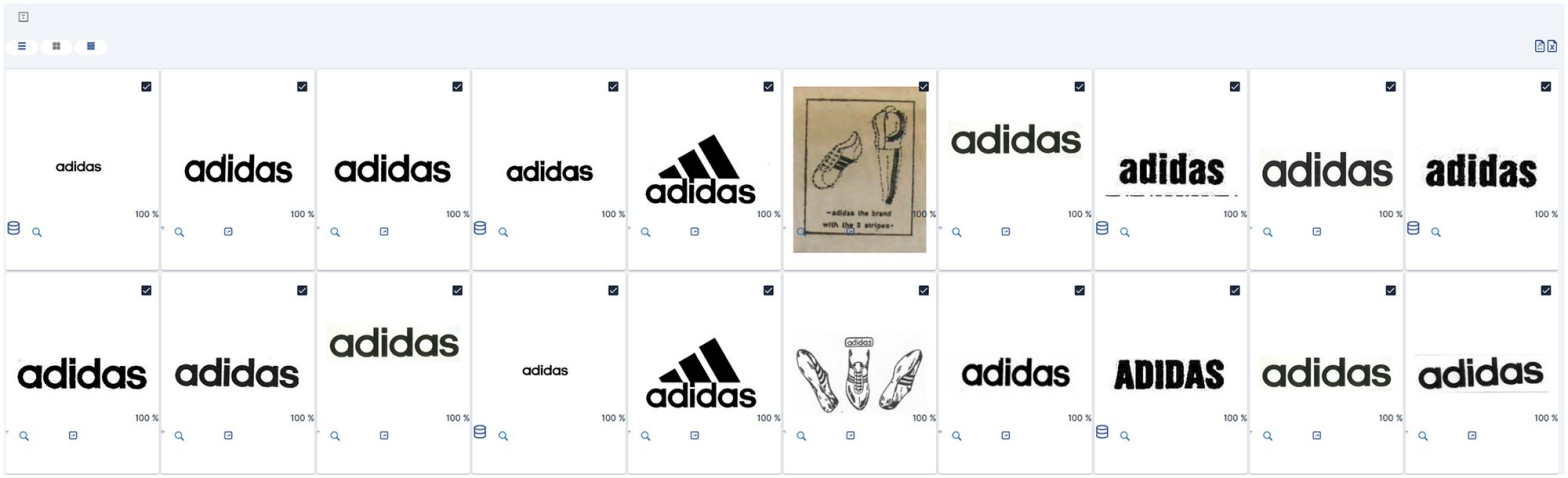
Sample C—Text-only search. Reproduced under a CC BY license, with permission from Techframe—Sistemas de Informação, SA, original copyright 2023.

Another distinguishing feature of our system is that submitting an image is, in fact, not necessary to perform high quality searches. Users can perform a text-only query with no graphical component and be presented with trademark images matching the provided text, as showcased in the example illustrated in Fig 15. For text-only searches, we rely on additional metadata attached to each process to perform a custom fuzzy string matching process which works for integral or partial string similarities. For this sample, we used a particularly recognizable name so that the results are familiar to more readers, and to showcase those multiple known variants of the brand’s trademark image were correctly returned.

### Semantically aware search

**Fig 16:**
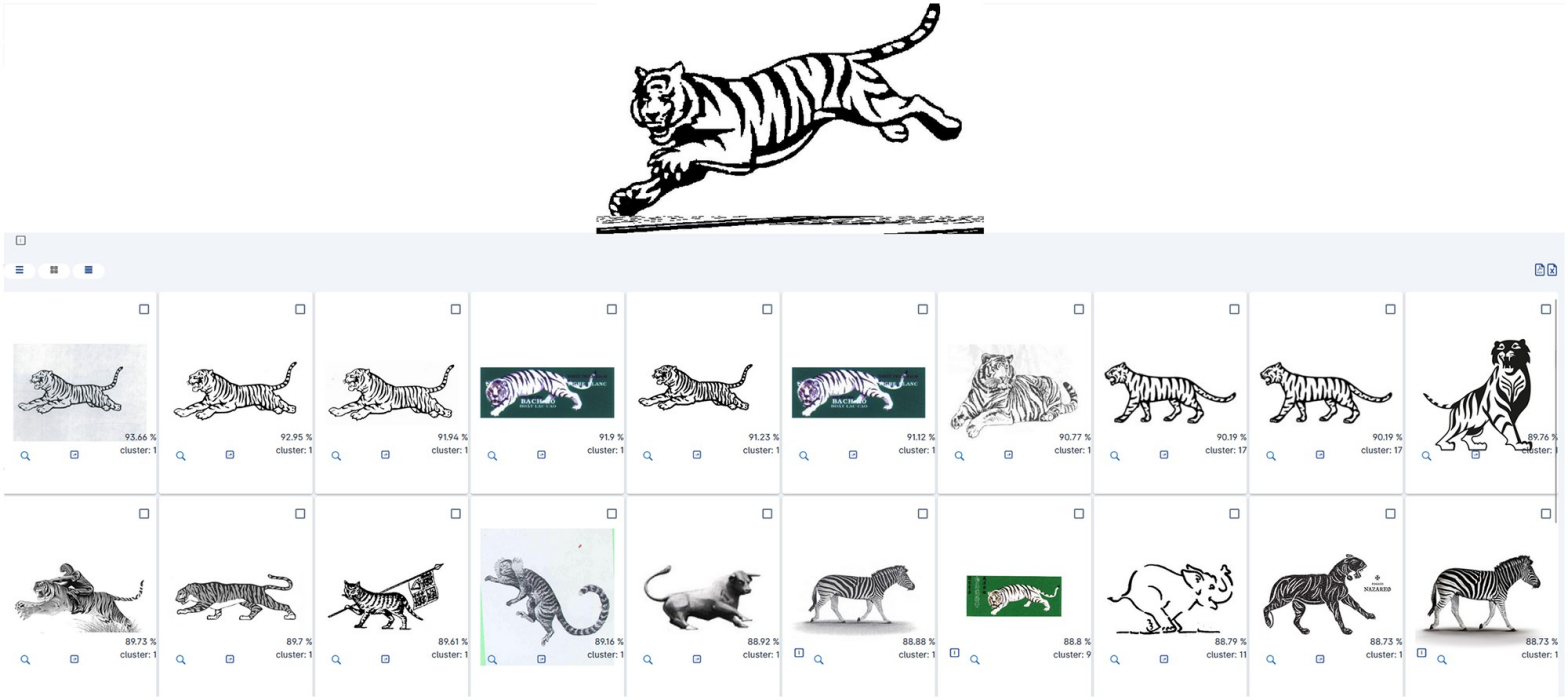
Sample D—Semantically aware search. Reproduced under a CC BY license, with permission from Techframe—Sistemas de Informação, SA, original copyright 2023.

Lastly, the example illustrated in Fig 16 provides a straightforward look into semantically aware searches. Here, the result set is almost entirely based on the action of sentence-encoders, which produce high-dimensional embeddings that essentially describe an image’s contents with tensor values (float numbers). Thus, using matching embeddings as the main search component often produces result sets with well represented concepts. In this particular example, most results either share an exact semantic concept (tiger) or represent other similar ideas (striped animals, four-legged mammals).

## 8 Scientific and technological relevance

The culmination of the aforementioned modules, and all the techniques applied therein, constitutes what we believe is a significant breakthrough in the IP sector, providing a solution to many of the problems highlighted previously. In summary, our system showcases timely retrieval speed, with 95% of searches falling within a 10 second presentation speed (sample size, *n* = 16530), and has a proven mean average precision (mAP) of **93.7%**. Given that data recentness if a top development priority, we are pleased to conclude that our growth module showcases a latency of less than **12 hours** between the moment a new trademark enters our knowledge base and the instant it can be searched. The recurring processing module has been designed to overshoot the expected daily trademark inflow and thus has an effective efficiency rate of approximately **1880%**, prospected to stay relevant and sufficient for years of increasing demand. We believe that our system represents a technological evolution, allowing tangible gains in the efficiency of the algorithms and the quality of the results. Several contributions have been made to the state of the art regarding content-based image retrieval systems, where we highlight our proprietary object extraction engine, providing insights into various image processing techniques and a new image retrieval system from trademark-based multi-feature extraction and deep networks, providing additional context on deep learning techniques employed to enable comparison of blended features in the DarwinGSE system, revealing state-of-the-art performance in high variance unlabeled datasets.

## 9 Conclusion

In this era, we are witnessing an increasing digitization of the most different types of information, which can take simple forms, such as texts, or more complex ones, such as images and videos. If, on the one hand, technological advances allow easier access to a greater amount of information, on the other, the increase in databases translates into a challenge in terms of identifying information. In the field of Industrial Property, information in image format is particularly important, since it is through their brand images that companies quickly establish contact with their customers. For this reason, companies make great efforts to protect their brand images from violations that could compromise their unequivocal recognition by consumers. One of the mechanisms most used by companies to safeguard their brand images is graphic search, comparing them with those stored in large databases, with the aim of finding images with a high degree of similarity. Brand images have characteristics, such as content coverage and abstract composition, that makes information retrieval a challenging task. Despite the evolution of CBIR systems, there are notable problems still to be resolved, particularly with regard to efficiency and reliability, factors of extreme importance in the area of IP, where inaccurate results can lead to legal action.

In this paper, we exposed and detailed the most relevant development choices carried out to make a distinctive contribution to the IP protection industry. Our approach is funded by modern trends and best practices and benefits from a healthy cycle of processes designed to overcome the difficulties associated with having a highly diversified dataset, as well as expanding upon other, previously proposed concepts such as semantic evaluation and multi-feature weighing. In an era where user data is collected and traded as an asset, our system stands out for being capable of gradual growth and result optimization while recovering only basic usage information, maintaining privacy and keeping cookie-based data transfers to a minimum. Being a system distributed as a service, we foresee a near future where additional improvements are required to ensure our system stays relevant and is the top choice for users who wish to keep an informed tab on their IP assets. We have therefore begun preliminary work to further improve semantic recognition, with the aim of extending our system with an additional module capable of segmenting image regions into known concepts, using sentence encoders and dynamic classification dictionaries. This will eliminate the human error and time required to use the Vienna Classification to assign conceptual labels to trademarks.

## Abbreviations

The following abbreviations are used in this manuscript:

ABIR: Annotation-based Information Retrieval
API: Application Interface
BRISK: Binary Robust Invariant Scalable Keypoints
CBIR: Content Based Image Retrieval
CNN: Convolutional Neural Network
DCNN: Deep Convolution Neural Network
DGQN: Deep Generative Query Networks
DIR: Deep Image Retrieval
DRR: Deep Relevance Ranking
FAISS: Facebook AI Similarity Search
FAST: Features from Accelerated Segment Test
FREAK: Fast Retinal Keypoints
HOG: Histogram of Oriented Gradient
IP: Intellectual Property
IR: Information Retrieval
IRS: Information Retrieval Systems
LBP: Local Binary Pattern
PDC: Percentile Difference of Changes
R&D: Research and Development
RGB: Red, Green, Blue
SIFT: Scale-Invariant Feature Transform
STAR: System for Trademark Archival and Registration
SURF: Sped-up Robust Features
SVM: Support Vector Machine
WIPO: World Intellectual Property Office
